# Initial experience with the Pascal photocoagulator: a pilot study of 75 procedures

**DOI:** 10.1136/bjo.2008.139568

**Published:** 2008-07-24

**Authors:** C Sanghvi, R McLauchlan, C Delgado, L Young, S J Charles, G Marcellino, P E Stanga

**Affiliations:** 1 Manchester Royal Eye Hospital, Manchester, UK; 2 OptiMedica Corporation, Santa Clara, CA, USA

## Abstract

**Background:**

The Pascal is a semiautomated photocoagulator that delivers a pattern array of multiple burns in a rapid predetermined sequence with a single foot pedal depression. Each burn is reduced to 10 or 20 ms to achieve this. The authors report their early experience with this system.

**Methods:**

75 procedures done in 60 patients divided into four groups—group A, patients undergoing panretinal photocoagulation (PRP); group B, patients undergoing focal or modified grid macular laser; group C, patients undergoing macular grid and group D, patients undergoing retinopexy—were retrospectively studied.

**Results:**

31/34 procedures in group A, 24/26 procedures in group B, 5/7 procedures in group C and all eight patients in group D had successful outcomes. Significantly higher powers were required with the Pascal than with conventional laser (p<0.001) in eyes that underwent PRP and focal/modified grid macular treatment with both systems. Single session PRP was successfully performed in five patients, and five were successfully treated with a macular grid using pattern arrays only. No adverse events were noted.

**Conclusion:**

Although the shorter pulse duration of the Pascal necessitates the use of a higher power, it is not associated with adverse effects. The results here suggest that the Pascal photocoagulator is safe and effective, and offer several potential advantages related to the brief exposure time.

Laser photocoagulation remains the gold standard in the treatment of many retinal vascular disorders. Conventional photocoagulation using a single application of laser energy per shot is usually delivered as a 100–200 ms duration burn. The Pascal (Pattern Scan Laser) Photocoagulator, which received United States Food and Drug Administration (FDA) clearance in 2005, semiautomates the procedure by delivering, with a single foot pedal depression, multiple laser burns in a rapid predetermined sequence in the form of a pattern array produced by a scanner. To achieve this, the pulse duration of each burn is reduced to 10–20 ms. This retrospective pilot study aims to discuss our early experience with this system and to record the laser parameters, especially the power needed to produce therapeutic burns.

## METHODS

A retrospective case note review of patients who underwent Pascal treatment at Manchester Royal Eye Hospital between November 2006 and May 2007 was performed. Information was collected on age, sex, indication, pre- and postprocedure best corrected visual acuities (BCVA), need for subtenon’s anaesthetic as well as outcome and complications of treatment. Treatment parameters including use of a pattern or single spot, type of pattern, power, burn duration and number of burns per session were noted. If the same eye had been previously treated for the same indication with one of our conventional single spot lasers (HGM, Salt Lake City, UT and Litechnica, Manchester, UK), the power, numbers of burns, spot size and burn duration were recorded in an effort to compare the settings needed with each system.

Four groups were created: group A, patients undergoing panretinal photocoagulation (PRP) for proliferative diabetic retinopathy (PDR) and ischaemic vein occlusions; group B, patients undergoing focal or modified grid macular laser for macular oedema secondary to diabetic retinopathy and branch retinal vein occlusion (BRVO); group C, patients undergoing grid treatment for diffuse diabetic macular oedema; and group D, patients undergoing retinopexy for retinal breaks/degenerations.

Moderate intensity burns producing retinal blanching were used for PRP and retinopexy, while macular burns were lighter. Treatment for Group A was deemed successful if, at the latest follow-up visit, neovascularisation had regressed, and no further treatment was planned, for Groups B and C if the macula was dry and the oedema had resolved, and for Group D if the break/degeneration was well surrounded on all sides with an adequate reaction.

The Pascal is a 532 nm frequency-doubled neodymium-doped yttrium aluminum garnet (Nd:YAG) solid-state laser. It can deliver numerous patterns including squares, arcs, full and subset grids, the shapes and sizes of which are adjustable, in addition to single spots. For PRP, the 3×3, 4×4 and 5×5 arrays were most commonly used, but in four eyes, single spots were administered for the entire PRP. Whether single spot or pattern array setting was used was decided independently of clinical findings. Prior to starting treatment, the operator chose whether or not to use Pascal based on their experience with the use of the new Pascal system.

All burns were placed one burn width apart. Subset grids and single spots were used for focal macular oedema. The full macular grid pattern was used for five patients with diffuse macular oedema who had good fixation, but single spots were used for the rest. A combination of the arc pattern and single spots was used for retinopexy with arc radii between 2000 and 3200 μm. Groups A and D received 20 ms, 200 µm spot size in air using a contact lens with a spot-size magnification factor of 2× producing burns of approximately 350–400 µm on the retina. Macular photocoagulation was performed using 10 ms exposures and a spot size of 100 µm in air which produced a ⩽100 µm burn on the macula because a contact lens with a spot-size magnification factor of 1× was utilised. Laser uptake may vary within the same fundus due to contact lens curvature, refraction, eye curvature and tissue characteristics such as pigmentation, so power needs to be varied as with conventional lasers until the desired burn intensity is achieved. Though efforts were made to avoid previous laser burns by adjusting the location of the arrays as necessary or changing the array pattern, this could not always be achieved. No obvious side effects resulted from this conduct.

Data were analysed using SPSS (Statistical Package for Social Sciences V.13). Descriptive statistics were used to summarise data and explore groups. Visual acuities (VA) were converted from Snellen to logMar to explore changes in vision pre- to postlaser using the Sign and Kruskal–Wallis tests. For those patients in groups A or B who had both previous conventional laser and Pascal, differences between laser parameters were explored, particularly the power using T and Mann–Whitney U tests. A two-tailed p value of less than 0.05 was considered significant (0.95 level of confidence).

## RESULTS

In the study period, 121 procedures were performed, but a complete set of data was available in only 75 procedures done on 60 patients, of whom 32 (53.3%) were male, and 28 (46.7%) were female with a mean age of 58.9 years (SD 15.3, range 14–86). VA did not differ significantly pre- to postprocedure (p = 0.359) in any group. There were 34 (45.3%) procedures in group A, 26 (34.7%) in group B, seven (9.3%) in group C and eight (10.7%) in group D (table 1).

**Table 1:** Analysis of Pascal parameters, visual acuity and outcomes by group

	Group A	Group B	Group C	Group D
No. of procedures	34	26	7	8
Prelaser VA logMAR, mean (SD)	0.31 (0.23)	0.30 (0.24)	0.6 (0.61)	0.01 (0.19)
Snellen equivalent	6/12	6/12	6/24	6/6
Postlaser VA logMAR, mean (SD)	0.30 (0.27)	0.22 (0.24)	0.53 (0.61)	0.01 (0.19)
Snellen equivalent	6/12	6/9	6/18	6/6
Pascal power (mW), mean (SD)	389 (93.6)	135 (36.6)	154 (32.9)	332 (105.5)
Pascal no. of burns, mean (SD)	1180 (460.7)	78 (45.3)	73 (33.8)	159 (102.2)
Average follow-up in weeks, mean (SD)	10.8 (5.9)	9.8 (3.3)	8.0 (5.5)	6.3 (3.2)
Successful outcome	31/34	24/26	5/7	8/8

logMAR, logarithm of the minimal angle of resolution; VA, visual acuity.

**Figure 1:**
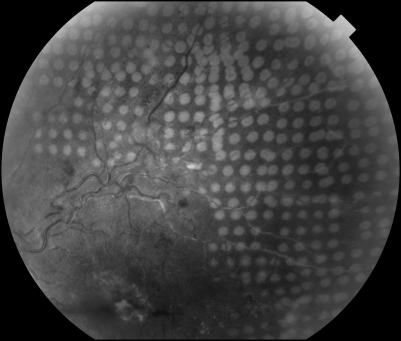
Colour fundus photograph showing uniformly distributed and homogeneous laser burns produced with the Pascal photocoagulation system in a patient who underwent sectoral laser photocoagulation for ischaemic branch retinal vein occlusion.

Of the 34 Group A procedures, 29 (85.3%) were for PDR, three (8.8%) for vein occlusions (fig 1), one (2.9%) for ocular ischaemic syndrome and one (2.9%) for proliferative sickle retinopathy (PSR). The average laser power, number of burns and mean follow-up period for the whole of group A are listed in table 1.

Of the 34 PRP procedures, 12 (35.3%) used only the Pascal photocoagulator. For the remaining 22 (64.7%), the Pascal episode was for additional fill-in PRP, as they had previously had conventional laser photocoagulation, but this had not adequately controlled the neovascularisation. This group therefore allowed us to directly compare the laser power needed using a 100 ms burn for the conventional treatment with the laser power needed for the same eye during the Pascal episode using a 20 ms burn. The average power with the conventional photocoagulator for these 22 procedures was 235 mW (SD 57.2, range 170–400), and the mean number of burns was 738 (SD 231.2, range 320–1178). The Pascal parameters used for these 22 procedures were as follows: mean power was 396 mW (SD 100.2, range 250–750), and the mean number of burns was 1116 (SD 417.2, range 500–2063). The difference in powers used with the conventional and the Pascal lasers for these 22 patients was highly significant (p<0.001), being 396 mW for Pascal compared with 235 mW for conventional laser. Though not specifically looked at in this study, it was generally acknowledged by operators that power had to be reduced when treating the peripheral retina in comparison with power used posterior to the equator.

Similarly, in these 22 patients, the mean number of burns given with Pascal (1116) was significantly greater than the mean number of burns given with conventional laser (738) (p = 0.004). Of these 22 procedures, 19 were successful with regression of neovascularisation at their latest follow-up visit. Three eyes needed further laser. Three patients had needed a subtenon’s anaesthetic for their conventional laser session, but none of them required it for their Pascal procedure.

Of the 12 PRP procedures done exclusively with the Pascal, five (41.7%) were performed in a single session, and the rest were fractionated into two episodes. The mean number of burns given during single session PRP was 1498 (SD 592.7, range 508–2069). None of the eyes with single session PRP developed any complications, and regression of neovascularisation was noted in all, with no further treatment planned at their last follow-up visit.

Group B included 26 procedures, of which three (11.5%) were focal treatments for macular oedema secondary to BRVO, and 23 (88.5%) were focal treatments for diabetic CSME. The average laser power, number of burns and mean follow-up period for the whole of group B are in table 1. Ten (38.5%) eyes had had previous focal macular laser in the same area using conventional laser with an average power of 100 mW (SD 21.6, range 70–140), spot size 50–100 µm and burn duration of 50–100 ms. The mean number of burns was 88 (SD 76.6, range 15–276). For those 10 patients who had previous conventional laser, significantly higher powers were used for Pascal (143 mw) than conventional (100 mw) (p<0.001) treatment. Following Pascal treatment, in 24 of the 26 procedures, the macula was dry, and no further laser was required. Two patients had residual CSME, of which one underwent further laser, and one had intravitreal triamcinolone acetonide injection (IVTA).

**Figure 2:**
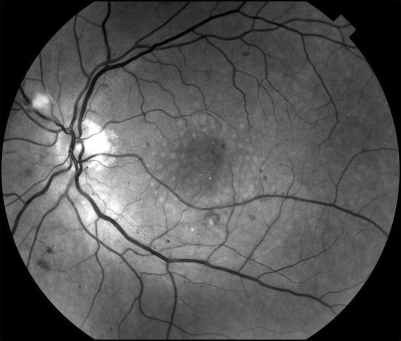
Red free fundus photographs of a macular grid applied using the Pascal pattern in diffuse diabetic macular oedema. Notice the evenly distributed burns.

In Group C, there were seven eyes of six patients who underwent a macular grid, five using the Pascal pattern (fig 2) and two using single spots. The average laser power, number of burns and mean follow-up period for the whole of group C are listed in table 1. Two eyes did not achieve resolution of macular oedema and underwent IVTA injection.

**Figure 3:**
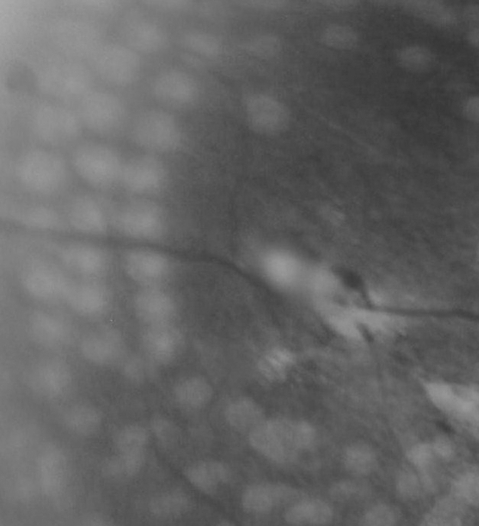
Retinopexy image.

Retinopexy (fig 3) was done using the Goldman 3 mirror lens. Application of an appropriately sized arc pattern was particularly advantageous, as one entire edge of the break could be sealed with a single footpedal depression as soon as a good view was obtained, making the procedure quick and easy. The treatment parameters are listed in table 1. All were successful with adequate laser reaction all around, and none needed further treatment.

Topical anaesthesia was sufficient in all groups, and none required any other anaesthetic. No complications related to laser treatment were noted in any patient. No effects were observed on blood vessels if the array inadvertently involved a retinal area traversed by blood vessels. None of the patients experienced bleeding of either retinal or choroidal origin. No effects were observed due to the operator being unable to avoid old laser burns in re-treatments. The results here reported are based on the data available. The procedures not included did not seem to differ noticeably from those presented.

## DISCUSSION

During photocoagulation, the aim is to optimise thermally induced therapeutic effect but cause minimal retinal damage. Laser–tissue interaction is influenced by wavelength, spot size, power and exposure time. Retinal damage can be reduced by changing some of these parameters. Pascal technology utilises an exposure time of 10 ms for macular photocoagulation and 20 ms for PRP. Our experience reveals that this brief exposure requires a higher power to achieve the desired therapeutic lesion. Twenty-two eyes undergoing PRP in group A (n = 34) underwent photocoagulation with conventional laser and Pascal. There was a highly statistically significant difference in the mean power used between conventional laser (235 mW) and Pascal laser (396 mW) (p<0.001). Similarly in group B, the mean power used was significantly higher with the Pascal system (143 mW) than with the conventional system (100 mW) (p<0.001). A prospective case controlled randomised clinical trial comparing laser parameters and outcomes between two groups, one undergoing Pascal laser and the other a conventional laser, would be the ideal way to highlight the differences in power utilised by the two systems. Until these results are available, our data give an indication of the higher power settings needed with the Pascal system as compared with conventional photocoagulation.

However, these higher power levels required with the Pascal system did not result in any complications. This may be a reflection of the reduced laser energy per burn reaching the eye secondary to its shorter duration. Fluence is calculated as power×time/area, and provided that spot size remains unchanged, with a burn duration of 20 ms the fluence is less than with a 100 ms burn when titrating to the same burn intensity because of reduced diffusion of heat. In a histopathological study of rabbit eyes comparing the effects of various pulse durations and powers, the power required to produce ophthalmoscopically visible spots decreased with increasing pulse duration, but the cumulative pulse energy increased with pulse duration.^1^ In conventional photocoagulation, a 400 µm spot will produce a burn with a diameter greater than 400 µm, whereas with a 20 ms pulse duration, the burn diameter will be less than the spot diameter. While the energy per pulse is reduced, the total energy delivered to the retina may be the same because more spots must be delivered to compensate for smaller burns.

There has been some concern that very short exposures may cause acoustic shock wave damage and haemorrhage. Some early argon laser studies showed a narrow safety margin between retinal burn and retinal haemorrhage for pulse durations less than 50 ms.^2^ ^3^ It has been since then shown that the point of change from thermomechanical cavitation-induced RPE damage to pure thermal RPE denaturation occurs at a 50 μs exposure time, a much shorter time than that employed by the Pascal system. At pulse durations longer than 10 ms, pure thermal denaturation of tissue is the primary retinal damage mechanism.^4^ ^5^ It is this thermal effect that produces therapeutically desirable retinal lesions.^6^ In the same histopathological study of rabbit eyes cited above,^1^ using pulse durations of 20 ms, the threshold for a visible burn was 110–120 mW, while that for retinal haemorrhage was 600 mW, suggesting an adequate safety margin. Another recent study on rabbit eyes has demonstrated that 20 ms pulse durations represent an optimal compromise between reduced collateral damage and sufficient width of the therapeutic window.^7^

Regression of neovascularisation is associated with greater areas of retinal ablation at the initial treatment session.^8^ The mean number of burns used for single session PRP with the Pascal was 1498, of which one patient had ischaemic BRVO which needed only 508 burns. Resolution of neovascularisation is significantly related to the cumulative total number of burns, and successful photocoagulation requires considerably more treatment than suggested by earlier studies.^9^ With conventional photocoagulators which deliver spot-by-spot treatment, this has to be balanced against patient and operator discomfort associated with the longer time required to achieve greater retinal ablation per session. The array method of multiple burn application allows for a larger area of retinal ablation in a shorter time. However, although single-session PRP may be possible with the Pascal system, its feasibility may be debatable due to concerns such as macular oedema and exudative retinal and choroidal detachments. In a small randomised trial by Doft and Blankenship, these did occur more in the first few weeks after single-session PRP, but the effects were transient, and no long-term difference between single and multiple session treatment groups was found.^10^ In our patients, none who underwent single-session PRP had any complications, but the numbers were too small to draw a conclusion.

Longer burns may cause greater thermal diffusion, whereas for short pulse durations, there is minimal diffusion of heat to adjacent areas, resulting in localised homogenous burns.^11^ This may theoretically result in less discomfort. With topical anaesthesia only, some patients find it difficult to tolerate 100 ms burns necessitating subtenon’s or peribulbar anaesthesia.^12^ In our series of patients, this was not required. Three patients who had previously undergone PRP using 100 ms burns required subtenon’s anaesthetic for those procedures but were able to tolerate the Pascal procedure with only topical anaesthesia. A recent study has shown that shortening the exposure time to 20 ms is significantly less painful but equally effective as conventional parameters.^13^ Reducing the burn duration combined with the application of a grid pattern has the potential of reducing overall treatment duration, thereby reducing costs to both hospitals and patients. Due to the retrospective nature of our study, actual treatment times and pain analogue scales were not available for analysis, but prospective studies are under way in our unit to evaluate the same.

This study, being retrospective and non-randomised has limitations but justifies the need for prospective larger trials comparing the Pascal technique with conventional lasers. Innovative methods of improving upon the precision, safety, comfort and efficiency of photocoagulation procedures are welcome, and our early experience seems to indicate that the Pascal method can offer this opportunity.
